# Risk factors and coping strategies of music performance anxiety among student pianists in higher education: a phenomenological research perspective

**DOI:** 10.3389/fpsyg.2026.1804936

**Published:** 2026-06-25

**Authors:** Xiaoyu Miao, Xinyue Wang, Ahmad Faudzi Musib

**Affiliations:** 1 College of Music and Dance, Xihua University, Chengdu, Sichuan, China; 2 The Graduate School Arts & Culture, Sangmyung University, Seoul, Republic of Korea; 3 Faculty of Human Ecology, Universiti Putra Malaysia, Serdang, Selangor, Malaysia

**Keywords:** music performance anxiety (MPA), lived experiences, risk factors, coping strategies, tertiary student pianists

## Abstract

**Introduction:**

Music Performance Anxiety (MPA) is prevalent among tertiary piano majors and harms their performance quality and physical and mental wellbeing. Most existing MPA studies use quantitative methods, which fail to capture implicit risk factors and individualized coping experiences. Given the lack of systematic MPA intervention in Chinese higher music education, this study adopts qualitative research to explore MPA risk factors and coping strategies among Chinese tertiary piano students, providing targeted insights for MPA intervention.

**Methods:**

This study adopted a descriptive phenomenological design, using tertiary student pianists from 10 higher education institutions in China as research participants. Purposive sampling was employed to recruit 29 participants who met the inclusion criteria. Data were collected via one-on-one interviews and focus group discussions, and analyzed using Moustakas' modified Stevick-Colaizzi-Keen (SCK) phenomenological approach for thematic coding.

**Results:**

The research findings yielded eight core themes. Of these, four pertain to the risk factors of MPA, namely psychological factors, personal growth factors, piano performance-related factors, and excessive outcome expectations. The remaining four relate to coping strategies: self-psychological regulation, understanding and social support, practice makes perfect, and leveraging MPA to enhance piano performance.

**Conclusion:**

Based on the Social Cognitive Theory (SCT), this study for the first time systematically reveals the risk factors and coping strategies of MPA among tertiary student pianists in Chinese universities. It addresses the gap in qualitative research within the local context of this field, provides empirical support for the application of SCT in music education and mental health domains, and offers theoretical and practical insights for the identification, assessment, and intervention of MPA in higher education settings.

## 1 Introduction

Music Performance Anxiety (MPA) is a significant and persistent anxiety experience related to music performances, triggered by specific anxiety-conditioning experiences. It manifests through a combination of emotional, cognitive, physical and behavioral symptoms, and is prevalent among musicians of all ages, genders, performance experience and technical proficiency (Kenny, 2011). To date, there is no fully unified academic definition of MPA, yet the most comprehensive and accurate definition acknowledged by most scholars is: “music performance anxiety is the experience of marked and persistent anxious apprehension related to musical performance that has arisen through specific anxiety conditioning experiences. It is manifested through combinations of affective, cognitive, somatic and behavioral symptoms and may occur in a range of performance settings” (Kenny, 2010).

As a common psychological phenomenon in the field of music performance, MPA not only leads to a decline in performance quality and the premature end of musical careers, but also diminishes musicians' overall wellbeing (Ryan and Andrews, 2009). In severe cases, it can even exert long-term adverse effects on performers' physical and mental health, such as triggering headaches, nausea and shortness of breath, and increasing the risk of cardiovascular conditions, and even inducing anxiety disorders (Barros et al., 2022; Papageorgi, 2022). Within the higher education context, music students, particularly those specializing in piano performance, are at heightened risk of MPA due to their frequent exposure to high-pressure evaluative scenarios including solo recitals, professional assessments and academic reviews (Lupiáñez et al., 2022). Compared with professional musicians, tertiary student pianists are in a critical phase of technical skill development and psychological maturation, and are thus more vulnerable to the negative impacts of unfavorable feedback on their self-confidence and emotional health, with their levels of MPA typically being more pronounced (Sternbach, 2008; Farley and Kelley, 2023).

In the Chinese higher education system, piano performance education places strong emphasis on technical training and performance practice, yet no systematic framework exists for the intervention of MPA (Cui et al., 2024; Yang et al., 2022). Most existing research focuses on MPA within the context of Western music education, and research specifically addressing piano students in Chinese higher education institutions remains relatively scarce. Notably, there is a lack of in-depth exploration into the risk factors and coping strategies of MPA for this group through qualitative research methods. Descriptive phenomenology, as an effective research approach for exploring individual subjective experiences, can accurately capture the genuine feelings, cognitive patterns and behavioral responses of piano students when MPA manifests, thereby providing a key perspective for understanding the uniqueness of this cohort.

MPA arises from the interaction of multiple factors. At the individual level, gender, age, personality traits and perfectionism tendencies all exert significant impacts on MPA levels. Studies have shown that the incidence and intensity of MPA among female musicians are typically higher than among males (Guven, 2017; Wehr-Flowers, 2006). Young musicians and student groups are more likely to suffer from anxiety due to insufficient performance experience and underdeveloped psychological adjustment skills (Farley and Kelley, 2023). Perfectionism, particularly maladaptive perfectionism (e.g., excessive self-criticism and cognitive dissonance), is a well-documented key predictor of MPA, as it intensifies negative self-evaluation and fear of failure, thereby exacerbating anxiety during performances (Butković et al., 2022; Dobos et al., 2019). At the psychological level, the interaction between trait anxiety and MPA cannot be overlooked. Individuals with high trait anxiety are more prone to experiencing MPA in high-pressure situations such as performances, and Generalized Anxiety Disorder (GAD) acts as a mediating variable that plays a central role in linking MPA to other anxiety types (Wiedemann et al., 2022; Kenny, 2009).

Environmental and situational factors also exert a significant impact on the MPA experienced by tertiary student pianists. The evaluative pressure of performance contexts (such as audience presence, judge scrutiny and examination atmospheres), perceived technical difficulty of the repertoire, and performance mindset all significantly elevate MPA levels (Osborne and Kenny, 2008; Papageorgi, 2021). Within the educational environment, teachers' attitudes, feedback styles, quality of teacher-student relationships and peer support systems directly influence students' emotional regulation and approaches to coping with MPA (Tahirbegi, 2022). Moreover, cultural background, as a potential influencing factor, may indirectly affect the manifestation and intensity of MPA by shaping performers' self-perception, evaluation criteria, and social expectations (Perdomo-Guevara, 2014). This underscores the necessity of conducting localized research on MPA among student pianists within the Chinese cultural context.

In addressing the challenges posed by MPA, musicians have developed various coping strategies, including cognitive intervention, behavioral intervention, and supplementary therapies (Kenny, 2009; Ory, 2022). At the cognitive level, individuals can regulate cognitive evaluation to effectively reduce anxiety experiences (Spahn, 2015; Huang and Yu, 2022). At the behavioral level, increasing practice time, conducting mock performances, and learning relaxation techniques (e.g., deep breathing, muscle relaxation) are common coping strategies among student groups (Tahirbegi, 2022; Kenny and Halls, 2018). Supplementary therapies such as mindfulness training, meditation, and yoga have also been shown to alleviate MPA positively (González et al., 2018; Irie et al., 2023). However, the applicability of these coping strategies varies across individuals, and the strategies chosen by Chinese student pianists may be shaped by local factors, including educational models and cultural norms. The effectiveness and uniqueness of these strategies therefore need to be explored through qualitative research.

### 1.1 Theoretical framework

This study adopts Bandura's Social Cognitive Theory (SCT) as its core theoretical framework. The theory's central tenet, Triadic Reciprocal Determinism, posits dynamic interactions among an individual's cognitive, behavioral and environmental factors, which jointly influence the generation and regulation of human behavior (Bandura, 1986, 1991). Key constructs including self-efficacy, self-regulation and outcome expectation provide a systematic analytical perspective for examining MPA among Chinese tertiary student pianists. From the SCT perspective, the formation and management of MPA are the product of reciprocal interactions among personal, behavioral and environmental factors. Among individual factors, self-efficacy and self-regulation are the primary internal variables shaping participants' MPA experiences (Bandura, 1998, 2004). Behavioral factors are reflected in tertiary student pianists' piano performance practices and their coping behaviors in relation to MPA. Outcome expectations, encompassing expectations for performance standards and predictions regarding the efficacy of corresponding coping measures, may either support or hinder student pianists (Bandura, 2004). Environmental factors encompass social contexts such as university, family and daily life, as well as objective physical environments including performance venues and practice conditions. Such factors may act either as inducers of MPA or as sources of supportive resources for coping with MPA (Bandura, 2001).

**Figure 1:**
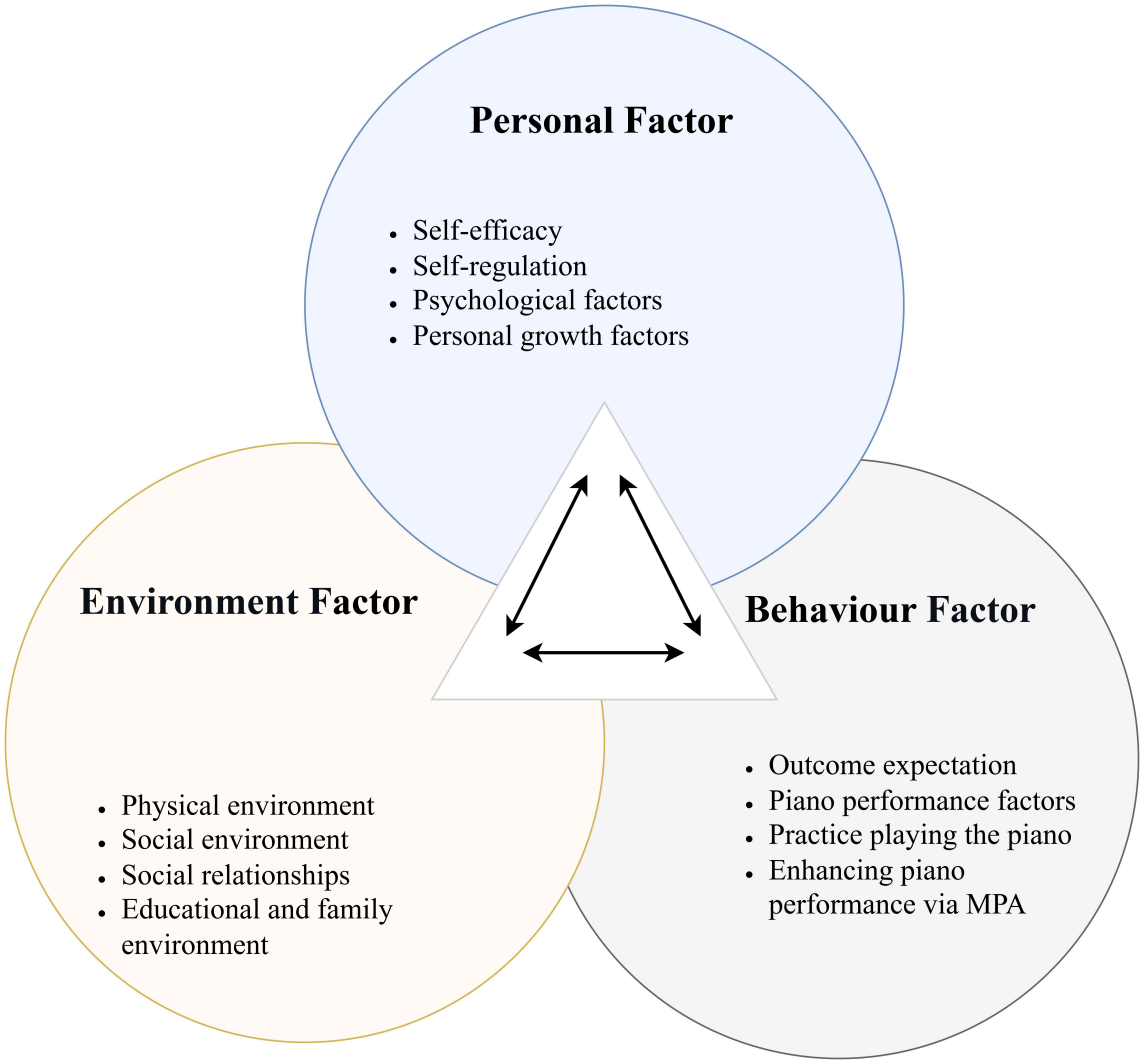
Theoretical framework of this study.

This theoretical framework is compatible with the present study because it integrates risk factors across personal, behavioral, and environmental levels, while also providing theoretical support for understanding the MPA coping strategies adopted by tertiary student pianists. It is particularly well-suited to revealing how student pianists, within the high-pressure context of Chinese higher education and against the backdrop of Chinese cultural norms, dynamically adapt through personal cognitive adjustment, behavioral strategy modification, and the use of environmental resources to cope with MPA (Bandura, 2001; Quyam and Abumehdi, 2022). Figure 1 outlines the theoretical framework adopted for this study.

### 1.2 Present study

Against this backdrop, current research still has significant gaps. Firstly, there are relatively few studies focusing on MPA among student pianists in Chinese higher education institutions, making it difficult to reflect the group's characteristics within the local educational context. Secondly, while quantitative research can reveal correlations between variables, it struggles to deeply capture the subjective experiences and dynamic processes of musicians' interactions with MPA and also fails to identify some personalized and less frequently discussed factors and coping measures. Thirdly, explorations of MPA risk factors mostly remain at a single dimension, lacking systematic analysis of interactions among multiple factors, including individual student traits, the educational environment, and social and cultural backgrounds. Fourthly, research on coping measures has not fully focused on the autonomous exploration and practical wisdom of the student group.

Based on this, this study employs a descriptive phenomenological approach, focusing on student pianists in Chinese higher education institutions, to conduct an in-depth investigation into the MPA-related risk factors and coping strategies of these students. Its aim is to address gaps in existing research, provide a theoretical foundation and practical guidance for the identification, prevention, and intervention of MPA in Chinese higher music education, and help student pianists maintain their physical and mental wellbeing and professional progression. This study also aims to provide empirical support for constructing an MPA intervention system that aligns with the local context. The core research questions of this study are as follows:

- What are the specific manifestations and subjective experiences of MPA among Chinese tertiary student pianists?
- What are the main risk factors leading to the emergence and exacerbation of MPA among Chinese tertiary student pianists?
- What coping strategies and methods do Chinese tertiary student pianists adopt to manage MPA?

## 2 Materials and methods

### 2.1 Research design

This study is a qualitative study that explores music performance anxiety (MPA) risk factors and the coping strategies adopted by participants, framed from the lens of their lived experiences. Moustakas (1994) proposed that the research questions best suited to phenomenological inquiry focus on exploring the common experiences of multiple individuals regarding a specific phenomenon. Creswell and Poth (2016) further noted that descriptive phenomenology enables researchers to examine specific experiences or phenomena from the standpoint of participants' lived experiences, and this approach aligns with the research objectives of the present study as it prioritizes the participants' subjective perceptions and authentic experiences without pre-imposed assumptions. This study therefore adopts a descriptive phenomenological research design. By detailing participants' MPA-related lived experiences, it aims to gain an in-depth understanding of the underlying causes of their MPA experiences and their practical coping strategies, and to uncover factors and strategies that may be overlooked in quantitative research, thus supplementing and enriching the empirical evidence base within this research domain.

### 2.2 Participant and recruitment procedure

When a study requires collecting information from the participants' perspective based on their specific characteristics, purposive sampling is the most effective sampling method (Campbell et al., 2020). Therefore, this study adopted purposive sampling to recruit participants. Following ethical approval, the first and second authors contacted the faculty members from piano departments of 10 Chinese higher education institutions. After informing them of the research purpose and procedures, and securing permission from their respective universities, these faculty members assisted in distributing the questionnaire to eligible students. Several questions in the questionnaire were selected from the Performance Anxiety Inventory (PAI) and Spielberger's State-Trait Anxiety Inventory (STAI), while others focused on the participants' basic information and open-ended questions related to MPA (Bascomb, 2019; Spielberger et al., 1971). The questionnaire was designed to understand the basic situation of tertiary student pianists in relation to MPA and to identify potential respondents. Since the questionnaire data bear little relevance to the research themes, they will not be presented or discussed in the subsequent sections.

The researchers collected 223 valid responses and selected 37 potential respondents based on the information provided in the questionnaire and the predefined inclusion criteria. After introducing the study purpose and data collection methods to these respondents, the researchers assured them that all data would be used exclusively for this study and informed them of their right to terminate or withdraw from the study at any time. Following a further understanding of the potential respondents' basic information and participation intentions, 29 participants who fully met all inclusion criteria and could provide rich, in-depth lived experiences were finally selected. To minimize potential sampling bias, participant screening was conducted independently by two researchers; any disagreements were resolved through discussion until consensus was reached. The sample was also designed to cover a range of ages, genders, study levels, and years of piano learning to ensure heterogeneity and enhance representativeness.

The inclusion criteria were defined as follows: (1) being a tertiary student majoring in piano performance; (2) having experienced music performance anxiety (MPA) in real performance situations; (3) being willing to share their lived experiences of MPA; (4) having used certain coping strategies to manage MPA; and (5) being able to provide fluent and in-depth descriptions. The adequacy of the phenomenological research sample was determined by data saturation. Some phenomenological scholars suggest that the optimal number of participants ranges from 6 to 12, and data saturation may be achieved after interviewing the 8th participant (Smith, 2003; Shorey and Ng, 2022; Boddy, 2016). Data saturation in this study was achieved after interviewing the 10th participant and the first Focus Group. Subsequent interviews and discussions only supplemented relevant details and further corroborated existing data, and no new themes emerged. This therefore confirmed the adequacy of the sample size. The basic information of the participants is shown in Table 1.

**Table 1:** Demographic profile of all participants.

Items	Variable	Range	Percentage
Age (years)	Minimum	18	/
	Maximum	28	/
	Median	23.14	/
Gender	Male	13	44.83%
	Female	16	55.17%
Educational background	Bachelor	21	72.41%
	Master	8	27.59%
Piano playing years	Less than 1 year	0	0%
	2–5 years	6	20.69%
	6–10 years	13	44.83%
	More than 10 years	10	34.48%

### 2.3 Data collection

This study collected data through one-on-one interviews and focus group discussions. One-on-one interviews enabled researchers to conduct in-depth discussions with participants about their MPA-related experiences, while focus group discussions offered an opportunity to explore the group's shared experiences. The combination of these two methods provided a more comprehensive perspective on the participants' experiences for this study (Shorey and Ng, 2022). Both interviews and discussions were conducted in a semi-structured format. Based on the participants' preferences, a total of 13 participants were interviewed individually, and 16 took part in focus group discussions. To avoid preconceived biases, focus group members were randomly selected. Focus Group 1 comprised 8 members, and Focus Group 2 also had 8 members. Based on the research objectives, the SCT framework, and the literature review, the researchers initially developed 11 general questions related to personal, social, and environmental factors as the interview guide (the interview guide is available in Supplementary Material 1). Before formal data collection, the researchers conducted pilot interviews with five tertiary student pianists not in the study group. Based on feedback from the pilot interviews, they revised and finalized the interview guide. The researchers distributed informed consent forms to the participants and began formal interviews after receiving their signed informed consent forms.

This study employed purposive sampling to recruit participants, conducted in accordance with the Declaration of Helsinki and following ethical approval from the Ethics Committee for Research Involving Human Subjects, Universiti Putra Malaysia. Interviews and discussions were conducted face-to-face between February 2025 and August 2025. Each interview lasted between 36 and 79 min, while the two focus group discussions lasted 97 min and 83 min, respectively. The first author served as the main interviewer, while the second author was responsible for audio recording, taking on-site notes, and providing further supplements or clarifications when necessary. Before data collection commenced, the first author introduced the significance, purpose, data collection procedures, and potential risks (i.e., recalling and describing MPA-related experiences might cause psychological discomfort) of the study to the participants. The researchers determined the timing and location of the interviews and discussions based on the participants' preferences and circumstances, with sessions held in electronic piano classrooms, piano rooms, the school meeting room, and coffee shops, respectively. Nine participants were interviewed twice, one participant three times, and the remaining participants, along with the two focus groups, had one session each. Additionally, the researchers maintained multiple follow-up communications with participants via social software (e.g., WeChat) to ensure the accurate and comprehensive collection and clarification of data.

Questions in the interview guide were appropriately expanded, revised, added to, or deleted based on each participant's actual situation to obtain more comprehensive and in-depth research data (Kallio et al., 2016). Formal data collection concluded when the first and second authors believed all relevant questions pertaining to the research topic had been addressed, the emerging themes could effectively support the research questions, and the participants confirmed they had fully shared their MPA-related experiences. Following participants' consent, the second author audio-recorded the entire process and took preliminary written notes. The first and second authors each completed verbatim transcriptions; the mutually cross-checked transcripts were then returned to participants for clarification and confirmation to ensure data accuracy and comprehensiveness. All audio recordings, transcripts, and participants' basic information are stored in an encrypted document on the first author's computer, and are used exclusively for academic research purposes. The audio recordings will be destroyed upon completion of the study, and no individuals other than the first author have access to the relevant information. To protect participant privacy, all one-on-one interview participants were assigned pseudonyms P1, P2,..., P13 in the order of their interviews, and the two focus groups were designated as FG1 and FG2 in this study. No personal identity information of any participant will be disclosed in the study's findings.

### 2.4 Data analysis

This study adopted Moustakas's modified Stevick-Colaizzi-Keen (SCK) phenomenological analysis approach as the framework for data analysis, with qualitative data analysis software employed for coding (Moustakas, 1994). Table 2 presents the specific analysis steps of the Stevick-Colaizzi-Keen (SCK) method.

**Table 2:** Data analysis steps of Stevick-Colaizzi-Keen (SCK).

Steps	Method
1	Familiarizing
2	Identifying significant statements
3	Converting significant statements into formulated meanings
4	Categorize meanings to develop themes
5	Exhaustive description
6	Producing the fundamental
7	Seeking verification for validation of findings

The first and second authors first transcribed all audio recordings verbatim into written documents. The two authors then cross-checked the transcribed documents and shared them with participants for clarification and verification, to ensure the accuracy and completeness of the transcripts. Given that participants lacked sufficient English proficiency for fluent communication, all interviews and discussions were conducted in Chinese. The authors subsequently translated the interview transcripts into English, with translation accuracy further verified via back-translation. The researchers then reviewed the transcripts repeatedly to familiarize themselves with each participant's MPA-related lived experiences. Open coding was then applied to code and categorize the data with the aid of qualitative data analysis software. Subsequently, the researchers extracted key statements pertaining to MPA risk factors and coping strategies from the coded data, and synthesized the underlying meanings of these important statements to develop themes related to the study's research questions. The example of this analytical process is presented in Table 3.

**Table 3:** Example of the Stevick-Colaizzi-Keen (SCK) phenomenological analysis process.

Significant statement (participant quote)	Formulated meaning	Core theme
“I kept thinking I would mess up, so I became more nervous and played worse.”	Negative pre-performance thoughts heighten anxiety and impair performance.	Psychological factors
“My family was very strict and expected me to play perfectly.”	High parental expectations contribute to persistent performance pressure.	Personal growth factors
“If I do not practice enough, I will definitely make mistakes on stage.”	Insufficient practice reduces confidence and increases anxiety.	Piano performance factors
“I care a lot about what teachers and others think of my playing.”	Self-worth is tied to external evaluation and social judgment.	Excessive outcome expectations
“I tell myself I can do it and focus only on the music.”	Positive self-suggestion helps stabilize emotion before performance.	Self-psychological regulation
“I practice difficult parts repeatedly until I am fully familiar with them.”	Targeted practice enhances mastery and reduces performance anxiety.	Practice makes perfect

Based on the participants' lived experiences, the researchers developed detailed, in-depth accounts of their MPA-related lived experiences in relation to the study's research questions. Then, the researchers deleted the repetitive, excessive, and unimportant descriptions in the overall structure, thereby focusing on the fundamental structure of the participants' lived experiences related to MPA risk factors and coping strategies. Finally, the researchers shared the transcripts and coding strips with the participants to verify whether they had accurately summarized and depicted the participants' MPA-related lived experiences.

### 2.5 Trustworthiness

As a phenomenological study, researchers served as “research instruments” throughout the entire process. To ensure methodological rigor, explicit bracketing (epoché) was practiced to set aside the researchers' prior experiences, preconceptions, and potential biases as piano educators. The first author systematically documented personal assumptions, expectations, and prior understandings about music performance anxiety in a reflexive journal before and during data collection and analysis. This reflexive practice helped prevent pre-existing beliefs from shaping participant responses or data interpretation, consistent with core principles of phenomenological inquiry (Moustakas, 1994; Lopez and Willis, 2004). The study was supervised by phenomenology experts and psychology practitioners to further enhance neutrality and reduce interpretive bias.

To maintain objectivity as insiders, several reflexive strategies were applied. Any personal assumptions or teaching experiences were explicitly bracketed and set aside during coding and theme development, ensuring that themes arose purely from participant data rather than researcher interpretation. Member checking was used to strengthen credibility. All participants were provided with summaries of the coded meanings and emergent themes to confirm whether the analysis accurately reflected their lived experiences. Participants verified or clarified interpretations, ensuring that the final themes remained grounded in their perspectives rather than researcher bias.

Furthermore, the trustworthiness of qualitative research should be evaluated across four dimensions: credibility, dependability, conformability, and transferability (Guba and Lincoln, 1994; Nowell et al., 2017). In this study, a questionnaire survey was first conducted to investigate the basic situation of MPA among Chinese tertiary student pianists. Under the framework of the SCT and SCK methods, data were collected and analyzed systematically to ensure the research's credibility, dependability, and conformability. Although this study only selected 10 Chinese higher education institutions as the research setting, the participants exhibited high homogeneity. Given that similar situations exist in other regions of China, the findings of this study can be transferred to other similar contexts. Finally, the Consolidated Criteria for Reporting Qualitative Research (COREQ) plays a crucial role in ensuring the credibility, transparency, and quality of qualitative research reports within the social sciences. This study employed a 32-item COREQ checklist to inspect and evaluate the entire research process, further ensuring the study's credibility (Tong et al., 2007; Buus and Perron, 2020). Further details are provided in Supplementary Material 2.

## 3 Results

This study aims to use qualitative methods to investigate the risk factors for music performance anxiety (MPA) experienced by tertiary student pianists through their lived experiences, as well as the corresponding coping strategies. The research results revealed four themes related to the risk factors, namely psychological factors, personal growth factors, piano performance factors, and excessive outcome expectations; and four factors related to the coping strategies, namely self-psychological regulation, understanding and support, practice makes perfect, and leveraging MPA to enhance piano performance. Table 4 summarizes the research themes.

**Table 4:** Emerging themes and transcripts samples.

Themes	Sub-themes	Transcripts samples	Occurrence
Risk factor	Psychological factors	“I had a concert in the evening, and anxiety was already there when I woke up that morning...”	10 interviewees 2 focus groups
	Personal growth factors	“My family is never felt proud of my playing or happy about it.”	8 interviewees 2 focus groups
	piano performance factors	“When performing pieces that I have not practiced enough, or when playing the first piece on stage, I would be extremely nervous and self-conscious.”	13 interviewees 2 focus groups
	Excessive outcome expectation	“Even when we are watching television, they (parents) ask me why I can not go on a world tour like Lang Lang. I really want to explode.”	9 interviewees 2 focus groups
Coping strategies	Self-psychological regulation	“When I adopted such a mindset, I gradually became more at ease on stage.”	9 interviewees 2 focus groups
	Understanding and support	“My teacher also helps us by adjusting our performances to minimize the impact of anxiety on our playing.”	10 interviewees 2 focus groups
	Practice makes perfect	“Ensure you practice extensively to develop a thorough grasp of the pieces you are to perform.”	12 interviewees 2 focus groups
	leveraging MPA to enhance piano performance	“I have discovered that mild MPA can spark a longing for the stage and an anticipation of the performance itself.”	8 interviewees 1 focus groups

### 3.1 Risk factors

#### 3.1.1 Psychological factors

Psychological factors were identified as a key risk factor contributing to participants' experiences of MPA. Participants exhibited an exceptionally high sense of self-efficacy in piano performance, which in turn drove them to pursue constant perfection. When their performances fell short of their own expectations, they would experience intense negative thoughts, which would trigger feelings of fear, worry, and anxiety regarding their subsequent performances. Consistent with comments from FG2, participants reported experiencing intense anxiety and low mood if they delivered a suboptimal performance in piano competitions or examinations:

“If I do not get a high score, I feel like I have let myself, my teacher and my parents down because I know I have the ability to get a high score. This feeling is really depressing” (One member).

“I agree. I think getting a high score in an exam or audition is a reward for me, so I strive for that” (Another member).

“For me, getting a low score in an exam or audition is really painful, because everyone else will scrutinize me and look at me with strange eyes. I feel absolutely terrible” (Another member).

Several participants mentioned that in the lead-up to a performance, they had constant negative thoughts and expectations, such as “I am sure to mess up”, “I will definitely forget my music when I go on stage”, “I will definitely make mistakes in the sections with difficult techniques”, “I will not be able to express my emotions on stage”, and “There will definitely be bad consequences” and so on. These thoughts could cause them to lose control of their mental and physical state due to anxiety during the performance.

“I had a concert in the evening, and anxiety was already there when I woke up that morning. When I was warming up, there was a voice in my head telling me I would definitely mess up at the concert. As time went on, I got more and more nervous and less confident. And sure enough, my performance was really bad in the end” (P13).

MPA prior to a formal performance could affect the participants' mental state, increasing the likelihood of them making mistakes during the performance. However, excessive anxiety during the performance would actually impair their performance quality, leading to errors, loss of control, and even performance interruption. As P2 stated:

“On stage, my mind was almost panicky and stiff. I did not know if I could finish the performance properly. I kept worrying I would make a mistake somewhere in the piece. Even though I tried to calm myself down and play like I usually do, things got more and more out of hand. My hands would involuntarily stray from the right notes, and my playing got worse and worse, even ridiculous. I felt so ashamed I wanted to crawl into a hole” (P2).

At the same time, poor performances in recitals, competitions, and examinations that stemmed from MPA could also have long-term psychological impacts on participants, exacerbating their subsequent MPA.

“After that disastrous performance, I started getting more and more afraid of playing in public, worrying about what would happen if I messed up or made a mistake” (P6).

“I realized MPA had turned into a vicious cycle. No matter how well I practiced or how thoroughly I prepared, once I stepped on stage, my mind went completely blank. My mindset fell apart, and I made more and more mistakes. I felt like I had to give up playing the piano” (P9).

Finally, two participants mentioned that the anxiety disorders they themselves suffered from would further exacerbate their MPA.

“Last semester, I was diagnosed with moderate anxiety. I am not sure if playing the piano caused my anxiety, or if the anxiety itself affected my playing. Anyway, even when I am not sitting an exam or giving a performance, just practicing on my own makes me feel anxious. The more I play, the more suffocated I feel, and my breathing gets shallower and shallower. I have to cool off first by drinking cold water and splashing cold water on my face, and only then do I feel a little better” (P7).

#### 3.1.2 Personal growth factors

As student pianists who had not yet officially established their social identities, their growth experiences also constituted hidden factors contributing to their MPA. Some participants grew up in high-pressure and unstable family environments, where there were tense family relationships, estrangement, personality conflicts, or physical disabilities among family members, causing them to lack a sense of security in life. Some participants were constantly criticized, blamed, or neglected during their growth, making it difficult for them to develop inner confidence. All these formative experiences could be the underlying causes of their MPA.

“My parents have worked away from home since I was a child, and I lived with my grandmother. Although they never shortchanged me materially, I had an emotionally lonely childhood. This left me with an introverted, conflicted and self-sabotaging personality. I am always worrying something bad will happen, and I think this is one of the reasons for my MPA. I also find myself worrying about making mistakes before a performance. I lack confidence in my playing and struggle with low self-esteem” (P3).

“My younger brother has autism, and my mum is always struggling with anxiety over it. She is a really fragile person, prone to feeling anxious, and she often ends up panicking and falling apart. I have witnessed her have countless emotional breakdowns—crying, yelling, even cursing us. I grew up walking on eggshells, and it is scary to realize my personality is a lot like hers. I have broken down and cried countless times during my piano lessons too. I just cannot shake the overwhelming anxiety I feel about performing...” (P10).

“My family has never felt proud of my playing or happy about it. They have always thought I should play as well as a professional pianist, and they would constantly say my playing was a mess, that they had made a mistake sending me to learn the piano, that I had no talent, and that my tuition fees were a total waste of money. After starting university, I tried to break free from this mindset, but those experiences have followed me everywhere...” (P5).

#### 3.1.3 Piano performance factors

Piano performance-related factors were the direct causes of the participants' MPA. Some participants stated that their MPA stemmed from inadequate pre-performance preparation and insufficient practice time, leaving them unconfident in performing the technically demanding sections of the repertoire and lacking the assurance to complete the performance successfully. Other participants noted that their psychological preparation was equally inadequate, and that they were unable to adjust their mental state to an optimal level. Consequently, they struggled to manage their nervousness, anxiety, and intrusive thoughts of fearing performance mistakes in the lead-up to and during performances.

“When performing pieces that I have not practiced enough, or when playing the first piece on stage, I would be extremely nervous and self-conscious. I was afraid that the audience would see through me and think that I was a lazy and technically poor pianist. The more I thought like this, the more anxious I became and the more prone I was to making mistakes” (P10).

“That was my worst experience. I felt that I had not prepared adequately, both in terms of performance and mindset. Before going on stage, I knew I was doomed” (P8).

Some participants mentioned that the performance environment could also induce their MPA. This environment encompasses both objective physical elements, such as unfamiliar competition, examination, and performance venues, pianos they had never played on before, and the venue's lighting, acoustics, and temperature and so on, as well as their subjective perceptions of the performance environment. For example, perceiving the competition's judges, audience members, and examiners to all be piano experts who would scrutinize their performance, believing their competitors to be more skilled than them, and feeling unable to command the stage with their own energy.

“At college, I practiced on Yamaha and Kawai. But for the exam, I had to play a Steinway piano, and I had never had the chance to play one before this. As soon as I stepped onto the stage, I felt nervous. The stage lights made me dizzy. In an instant, my heart tightened, and I could not find where to begin the first note” (P2).

“When I play in an unfamiliar environment, I get extremely nervous. I feel that I simply cannot handle any unfamiliar situation” (Focus group 1).

“When I arrive at the audition location, I feel that the piano is cold, and the keys are like ice. I even think that the audience below are unfriendly; they are waiting for me to make a mistake” (One member).

“I was taking an exam in front of several expert judges. I felt like a kindergarten child facing several doctors. I felt very embarrassed and inferior, thinking that I was worth nothing” (One member).

“Once, I participated in a piano competition. Before the competition, we were allowed to practice at the venue designated by the organizer. I heard that all my opponents were playing extremely well. Suddenly, I became nervous. As expected, I played terribly when I went on stage” (Another member).

#### 3.1.4 Excessive outcome expectations

The participants themselves held exceptionally high outcome expectations for their performance quality and their own piano playing careers. This served as the motivation for them to persist in piano practice and consistently participate in examinations, auditions, and performances. However, such overly high expectations also imposed a psychological burden on them, making them excessively anxious about their performance outcomes and leaving them excessively tense and apprehensive in the lead-up to and during a performance. As P10 stated: “The more I want to play well, the worse I perform; the more I worry about making mistakes, the more mistakes I make.”

“Every time I perform, I long for perfection. Unfortunately, in some cases, anxiety stops me from reaching my best level. I cannot do it...” (P1).

“When I was a child learning the piano, I dreamed of becoming a highly acclaimed pianist one day. I have always been very diligent in practicing and attending my piano lessons. But I feel that MPA has become a stumbling block in my piano career. I cannot get over it. Every time I give a public performance, I make mistakes to some degree” (P5).

Furthermore, overly high expectations placed on them by others could also be a contributing factor to their MPA. The participants' teachers and parents typically held exceptionally high expectations for them, hoping they would go on to become outstanding pianists in the future. Scrutiny from judges and experts who assessed them during examinations, competitions, and auditions also imposed an invisible pressure on them. Additionally, at concerts and performances, audiences and peers expected them to perform with ease and fluency like professional pianists. These external expectations could serve as motivation for them to refine their performance skills, but more often than not, they brought significant pressure and anxiety to the participants.

“My parents always compare me to other pianists. Even when we are watching television, they ask me why I cannot go on a world tour like Lang Lang. I really want to explode” (Focus group 1).

“Once, my professor recommended a performance for me. I played a piece by Liszt called ‘La Campanella'. When I got off the stage, I vaguely heard someone in the audience say, ‘The people from the conservatory of music cannot play any better than this.' This was a really big blow to me. Since then, I have become very afraid of public performances. Every time I go on stage, I am extremely anxious” (P9).

“My anxiety stems from the fact that I always attach too much importance to others' opinions. I long for recognition from others and gradually lose my own identity. When others say I play well, I feel happy. But when they say I play poorly, I feel as if I have no hope at all” (P4).

### 3.2 Coping strategies

#### 3.2.1 Self-psychological regulation

The participants would engage in their own psychological regulation through various means. They would offer themselves positive psychological suggestions, attempt to relax, and reduce negative and anxious thoughts ahead of going on stage using methods such as meditation and deep breathing. They would direct their attention to the performance itself and face it with a relatively relaxed and stable mindset. As P5 stated, ahead of going on stage, he would tell himself, “I have fully mastered and practiced this piece well. I can absolutely handle it. I only need to focus on my performance.” P4 also stated:

“Rather than just thinking ‘I want to perform a piece of music perfectly on stage', it would be better to think ‘I want to perform a piece of music to my own satisfaction on stage' or ‘I am merely sharing music with other people, not waiting for their judgment'. When I adopted such a mindset, I gradually became more at ease on stage” (P4).

“Before stepping onto the stage, I would meditate, imagining myself deep within a quiet forest or returning to my childhood. I imagined that it was my childhood self who was going to perform on stage, and she would only focus on how to perform this piece, not caring about anyone in the audience. In this way, I could avoid unnecessary and excessive thoughts and just focus on the performance itself” (P12).

Some participants mentioned that the alleviation of their MPA stemmed from the fact that they gradually built up confidence in performing through long-term psychological adjustment, and developed a strong sense of self-belief, believing they could successfully complete a performance.

“I have always been puzzled by the concept of ‘not practicing enough'. How can one determine when they have practiced enough? How long a practice session should last to be considered sufficient? And how can one measure the improvement in playing technique and emotional expression that comes with sufficient practice? Therefore, I believe that what can help overcome MPA is not merely the question of ‘enough practice', but a multifaceted confidence underpinned by a variety of factors” (P7).

“If I believe I can succeed in playing and hold this belief firmly, I will do so. This belief can overcome my psychological fears, my physical discomfort, and all the challenges associated with playing the piano” (P8).

However, when they no longer demanded that they never make mistakes during each performance or that they had to perform to their utmost at all times, and when they accepted the imperfections inherent in the performance process, their MPA diminished naturally.

“Accepting imperfection does not mean that perfection is a bad thing. It simply means that one has done their best. Even if a performance is imperfect, it is still a form of perfection. The key to a musical performance is whether the music is rendered beautifully, and whether the intended artistic elements are conveyed effectively. This is similar to life: it can be imperfect, but it needs to be beautiful” (P2).

#### 3.2.2 Understanding and support

Apart from self-psychological regulation, the understanding, guidance and support from others could also help the participants alleviate their MPA. Such understanding and support usually came from individuals who had similar experiences to the participants, including their piano teachers, fellow music students, and friends, as well as psychological professionals.

Some of the participants' piano teachers would share their own past performance experiences and the strategies they adopted to overcome MPA with them. They would tell the participants that MPA is a common experience among musicians, and that they need not be overly concerned about it. At the same time, the teachers would offer them professional encouragement and affirmation, as well as psychological empathy and comfort, thereby enabling them to gain greater strength to overcome MPA and build more confidence to pursue their piano studies and performance careers.

“My teacher often tells us that we are usually just passing visitors in other people's worlds. Even if we make mistakes on stage, after we step off stage, apart from ourselves, who else would remember them? Since adopting this mindset, I feel I have become fearless” (P10).

“My teacher also helps us by adjusting our performances to minimize the impact of anxiety on our playing. She says she fully understands our anxiety and has had the same experience, even having made mistakes on stage” (Focus Group 1).

Support from the participants' fellow music students and friends was equally important. As they shared similar experiences, they could more easily understand one another's circumstances. Through communication and sharing with their peers, the participants could receive encouragement, recognition and emotional comfort, and they could also exchange strategies for managing MPA with one another.

“My friends and I both have similar experiences and feelings. We often encourage each other, and we also work together to figure out ways to avoid making mistakes on stage. I am very grateful to have friends like these” (P9).

Some participants had experience consulting school counselors and seeing psychologists. Such professional psychological intervention was also highly beneficial.

“When I was at my lowest point and almost wanted to drop out of school, I gathered the courage to visit the school's psychological counseling room. analyze the source of my anxiety objectively and taught me anxiety relief methods for daily practice and performances, such as breathing adjustment techniques. Many of my classmates are still reluctant to consult psychological professionals now. I believe university could provide them with appropriate guidance” (P7).

“After a poor performance, my mother took me to see a psychologist. The doctor was very kind. She listened to my feelings gently and guided me through rational analysis of my situation. Although we only talked for less than an hour, I did feel much better afterwards” (P3).

#### 3.2.3 Practice makes perfect

Most participants stated that the most fundamental approach to managing MPA lay in undertaking long-term, thorough and effective practice prior to a performance. First, they believed that sufficient and prolonged piano practice was essential, as this allowed them to master the repertoire proficiently and develop complete assurance and confidence in their performance of it. Even if MPA arose on stage, their familiarity with the repertoire and well-honed muscle memory could mitigate its impact on their performance outcome.

“To play a piece of music well, I must master it to 200 per cent. If I have practiced to this standard, my MPA will diminish. Even if anxiety does arise, I can control it because I have prepared thoroughly. I can perform the piece with ease” (P12).

“Ensure you practice extensively to develop a thorough grasp of the pieces you are to perform. This can significantly alleviate psychological anxiety. After all, ‘practice makes perfect”' (P2).

Some participants also shared that overcoming MPA required not only undertaking long-term and extensive practice, but also attending to the adequacy and effectiveness of that practice. Proper practice and performance techniques, mastering technically demanding passages, calibrating one's performance mindset, and acclimatizing oneself to the performance environment, and the like, could not be overlooked. P5 and P11 shared their experiences in overcoming MPA by formulating detailed practice plans and refining their practice and performance techniques:

“Now I have formulated a detailed practice plan for myself. Before practicing the piano each day, I spend the first 2 h warming up, doing finger exercises, and focusing on targeted practice of the difficult sections within the pieces. I then practice the entire piece, concentrating on the musicality and completeness of the work, until I am satisfied with my playing before taking a break” (P5).

“Practice must involve the use of correct methods; such methods can help you overcome anxiety, avoid drawing a blank on the sheet music, and surmount technical obstacles. The most useful method for me is slow practice: listening to the left-hand melody, and concentrating on practicing the difficult sections. I also memorize the sheet music by dividing it into segments. For instance, splitting a single piece into five segments for memorization. In this way, even if I forget the third segment, I can continue playing from the fourth segment” (P11).

P3 stated that he would adjust his physical state during a performance, and that he would attain mental relaxation through bodily relaxation:

“When I am nervous, I always feel as if my body is rising up, and my heart is almost in my throat. So during a performance, I try to keep my body in a lowered posture, and to feel that my body is connected to the ground to form a single whole. Power flows from my back through to my fingertips. When my physical sensations are correct and I am relaxed, my mental tension is also greatly reduced” (P3).

Finally, some participants further stated that acclimatizing oneself to the performance environment ahead of a formal performance, including the performance venue, the piano to be played, and its accompanying facilities, could also help mitigate their MPA.

“If I have the opportunity to visit the performance venue before an exam or a performance, to familiarize myself with the surroundings and play on the piano there, I will not feel nearly as nervous during the actual performance” (P6).

#### 3.2.4 Leveraging MPA to enhance piano performance

Although the participants adopted various strategies for managing MPA, they stated that such anxiety could only be alleviated or kept within a manageable range, and that it could not be completely eliminated. It would resurface during subsequent performances. Therefore, some participants stated an additional coping strategy: harnessing MPA to refine their performance techniques and calibrate their performance mindset for upcoming performances.

P2 and P13 stated that they had learned to harness the tension and excitement elicited by manageable MPA to elevate their performance state, musical expressiveness, and playing quality during piano performance:

“As long as MPA remains within a controllable range, I can fully harness the excitement it generates. This excitement can enhance my concentration, allowing me to focus all my mental energy on the performance, and my expressive range will also grow stronger” (P2).

“If I were defeated by MPA, I would be a mediocre, untalented, and unaccomplished pianist. But if I channel this anxiety to elevate my playing, it can unlock my potential, enabling me to believe that I am capable of reaching a higher level of performance” (P13).

Some participants further stated that they would harness their MPA to calibrate their performance mindset during a performance, thereby elevating their playing quality:

“I have discovered that mild MPA can spark a longing for the stage and an anticipation of the performance itself. It helps me dispel the fatigue of daily practice and fill me with abundant energy, allowing me to deliver a higher level of performance during a concert” (P7).

“Having overcome that period, in which MPA took a toll on my physical and mental health as well as my playing ability, I resolved to let go of all burdens and be fully true to myself. First and foremost, I needed to build a strong sense of self, believing that my performance was of a good standard, and that being satisfied with it in my own right would be sufficient” (P8).

## 4 Discussion

This study was based on the triadic interaction determinism framework of Bandura's social cognitive theory (SCT) and adopted the descriptive phenomenological method to conduct an in-depth exploration of the risk factors and coping strategies for music performance anxiety (MPA) among piano majors in Chinese higher education institutions. It revealed the dynamic interplay between personal, behavioral, and environmental factors in shaping the participants' experiences and their manifestations of MPA. The research findings not only address the local research gap identified in the Introduction but also provide empirical support for the application of SCT in the field of mental health research related to musicians. Furthermore, by engaging with existing relevant literature, this study further enriches the understanding of cross-cultural differences and universal patterns of MPA. This section will discuss the findings from the three core dimensions of the SCT framework: personal factors, behavioral factors, and environmental factors, conducting a systematic analysis by integrating the research results, existing literature, theoretical foundations, and research implications.

### 4.1 Personal factors

This study's findings indicate that personal factors constitute significant risk factors for MPA among tertiary student pianists. The results show that the participants generally demonstrated a chain reaction characterized by “high self-efficacy—high outcome expectancy—heightened anxiety”. They held a strong desire to advance their performance techniques, which fostered an extreme pursuit of performative perfection. When their performances fell short of these expectations, negative automatic thoughts such as “I will mess up”, “I will be scrutinized” and “I will let others down” were triggered, which ultimately exacerbated their experience of MPA. These findings align closely with the core tenet of SCT that “personal cognitive factors govern behavioral regulation” (Bandura, 1991, 2004). Within the Chinese cultural context, collectivist values further strengthen outcome expectations, as students often link performance success to family honor and social approval. Perfectionism is reinforced by high external expectations, interacting with self-efficacy to amplify anxiety. These cultural and cognitive factors operate as key moderators within the SCT framework, shaping personal cognition and behavioral responses. This study also extends the work of (Guven 2017) by revealing that high self-efficacy does not always reduce MPA; instead, under strong outcome expectations, it may increase perfectionistic pressure and anxiety, offering a more nuanced understanding of the self-efficacy and MPA relationship. Furthermore, this study explored the experiences of Chinese student pianists with MPA-related anxiety, identifying trait anxiety as another contributing factor to the onset and exacerbation of MPA. This result echoes the research finding of Wiedemann et al. (2022) that individuals with high trait anxiety are more likely to trigger state anxiety in performance risk situations.

It is noteworthy that this study found that family environment and education during the participants' personal growth constituted underlying factors contributing to their anxiety tendencies. This finding expands previous MPA research by highlighting the long-term impact of early family experiences, which are underemphasized in Western studies but critical in the Chinese cultural context (Xie et al., 2008; Wong, 2024). Most participants recounted family-related experiences during their formative years, such as a lack of parental companionship, strained family relationships, and the physical or mental defects of family members, which all contributed to their anxious, sensitive, and tense dispositions. Furthermore, the family education these tertiary student pianists received underscored the uniqueness of personal factors within the Chinese cultural context. Consistent with Ma (2022) and Wang (2013), Chinese parents tend to hold high academic and performance expectations, emphasize educational investment, and link children's achievements to family honor, which increases perfectionism and performance pressure. Compared with those in other countries, the negative impact of growth experiences on Chinese tertiary student pianists is greater. This is partly due to the emphasis on obedience to authority in Chinese culture and the authoritative parenting of East Asian parents in families (Xie et al., 2008; Wong, 2024). In addition, Chinese tertiary student pianists often link their self-worth to others' evaluations. This tendency is deeply embedded in collectivist cultural values and concerns over social face in Chinese educational contexts. Students commonly view performance outcomes as a reflection of personal competence, teacher approval, and family honor, rather than purely individual achievement. As young pianists still developing a stable self-concept, they strongly need positive feedback and external approval. When such support is lacking, their vulnerability to music performance anxiety increases. The personality traits formed under these cultural and developmental conditions further elevate their likelihood of experiencing MPA (Quyam and Abumehdi, 2022).

Participants also alleviated MPA through positive psychological suggestion, meditation, and selfpsychological adjustment strategies. Essentially, this is grounded in the self-regulation mechanism of SCT (Bandura, 1998) and supports the view that cognitive restructuring can effectively regulate MPA (Spahn, 2015). The present study further extends this line of research by demonstrating that stable self-efficacy comes from integrated cognitive and emotional adjustment rather than technical proficiency alone, providing a more comprehensive target for MPA intervention. Finally, this study also found that MPA exerts not only negative effects on tertiary student pianists. Some participants stated that instead of striving to overcome MPA by all means, and failing to completely eliminate it, it is better to alter one's mindset and rationally harness the tension and excitement elicited by MPA to advance performance techniques and calibrate their performance mindset ahead of a performance, thereby allowing MPA to exert a controllable positive impact. This strength-based coping perspective goes beyond traditional MPA studies that focus only on anxiety reduction, offering a new theoretical insight for performance psychology.

### 4.2 Behavior factors

SCT posits that behavior acts as the intermediary between an individual and their environment. Personal cognition guides behavior and shapes environmental perception, while environmental feedback, in turn, regulates behavior and exerts a retroactive effect on personal cognition (Bandura, 1986). The findings of this study fully support this proposition. Piano performance is essentially a behavioral act, and it is also a key factor in triggering MPA. Insufficient practice, inadequate psychological preparation, ineffective practice methods, and other such factors can all induce MPA in the participants. This behavioral deficiency is fundamentally linked to personal cognition. The participants' vague perceptions of practice adequacy, such as the uncertainty of “what constitutes adequate practice”, leave them feeling anxious prior to performances due to a lack of confidence in their technical mastery. At the same time, the negative experience of a subpar performance acts as behavioral feedback that reinforces the negative cognition that “making mistakes on stage is inevitable”, thus forming a vicious cycle of “personal cognition—behavior—anxiety”. This aligns closely with Kenny's (2011) research finding that experiences of performance failure can exacerbate subsequent MPA.

At the coping strategy level, the study's findings indicate that the participants viewed long-term, intensive practice ahead of a performance as an important and practical measure. Moreover, most participants believed that efficient, purposeful practice, rather than simply increasing practice duration, could better alleviate anxiety. These findings confirm the positive regulatory role of behavioral factors in MPA. The participants enhanced their performance techniques and piece-playing proficiency by formulating detailed practice plans, optimizing their practice methods, and regulating their performance states. This not only refined their technical mastery and playing proficiency but also bolstered their self-efficacy through such behavioral efforts, forming a virtuous cycle of “effective practice—high self-efficacy—low anxiety”. This aligns closely with Tahirbegi's (2022) research conclusion that targeted practice strategies and simulated performances can significantly reduce students' MPA levels.

Furthermore, this study identified the unique anxiety management strategies adopted by Chinese tertiary student pianists. The participants recognized that MPA could not be fully eliminated and began to attempt to control MPA within a manageable range and harness this tension and anxiety to elevate their performance. This finding advances existing MPA research (Ory, 2022), which has mainly focused on reducing anxiety, by showing that students can instead transform mild anxiety into motivational energy for performance. The innovation of this finding resides in its breakthrough of the limitations of existing research, which has primarily focused on anxiety alleviation through coping strategies. This study is the first to propose a behavioral regulation strategy for the positive transformation of MPA, which aligns with Ory's (2022) proposition that coping strategies should also encompass emotional regulation and behavioral refinement.

It is important to highlight that the practice behaviors and coping strategies of Chinese tertiary student pianists demonstrate distinct local characteristics. Unlike their Western counterparts, the participants' practice strategies are typically structured and task-oriented. This is closely linked to the pedagogical model of Chinese higher music education, which emphasizes rigorous technical training (Yang and Welch, 2023; Wang, 2013). The practice behaviors shaped by this pedagogical model, while enhancing technical proficiency, may also intensify perfectionist tendencies owing to an overemphasis on normative standards. This further corroborates the core tenet of SCT that “behavior is the product of interaction between the individual and the environment” (Bandura, 1998).

### 4.3 Environment factors

According to SCT, environment factors serve as both the backdrop for behavior and a regulator of an individual's cognition (Bandura, 2004). This study's findings indicate that social and physical environments exert a significant influence on MPA among tertiary student pianists. On one hand, stress-inducing environments such as unfamiliar performance venues, judges' scrutiny, and others' high expectations constitute important risk factors for MPA. On the other hand, supportive environments such as teacher guidance, peer support, and professional psychological interventions act as key resources for alleviating MPA. Both types of environment interact with an individual's cognition and behavior, jointly influencing the intensity and manifestations of MPA.

In relation to stressful environments, the results of this study have identified two core factors that contribute to MPA among Chinese tertiary student pianists: physical environment discomfort and social evaluation pressure. This study first corroborates the findings of prior research (Osborne and Kenny, 2008; Papageorgi, 2021). The key theoretical extension of this study is the identification of the utilitarian educational atmosphere as a unique cultural stressor in Chinese piano education, which has rarely been addressed in previous international MPA research. This pattern is strongly supported by studies of Chinese parental involvement and educational culture (Ma, 2022; Wang, 2013), which highlight high parental investment, exam-oriented pressure, and outcome-focused evaluation. This finding reveals the invisible environmental influences that are difficult to capture in quantitative research, demonstrating the advantages of the phenomenological method (Bond and Petronzi, 2025).

In relation to supportive environments, this study's findings corroborate the tenet of SCT that “environmental resources can facilitate behavioral regulation” (Bandura, 1999). Tahirbegi (2022) reported that teacher-student relationships and peer support positively help music students manage MPA. Consistent with this, participants in this study received cognitive restructuring and encouragement from piano teachers through experience sharing and professional guidance. Peer support reduced their sense of isolation and self-denial, while psychological counselors offered professional interventions. Together, these supportive factors helped students relieve anxiety, adjust their mindset, and enhance self-efficacy. Notably, Chinese students relied more on teacher support, reflecting the cultural tradition of teacher authority (Tao et al., 2022; Pan and Yao, 2023). Teachers' feedback thus serves not only as professional guidance but also as validation of students' self-perception, making teacher support particularly critical for MPA regulation in the Chinese context.

## 5 Implications

This study deeply integrated social cognitive theory (SCT) into the field of higher music education and the Chinese cultural context. Through empirical data, this study verified the explanatory power of the tripartite interaction model for the phenomena related to music performance anxiety (MPA) among Chinese tertiary student pianists. It explored the risk factors and coping strategies for MPA from three dimensions: personal, behavioral, and environmental, thereby expanding the application scenarios of SCT. The significant strength of this study lies in its adoption of the descriptive phenomenological method and its grounding in China's local higher music education practice, filling the gap in qualitative research within the MPA field. Through in-depth exploration of the participants' subjective experiences, it not only revealed the MPA risk factors and coping strategies that are rarely addressed in quantitative research and possess local characteristics, but also identified novel findings such as “high self-efficacy inducing perfectionist anxiety” and “the utilitarian educational atmosphere as a unique environmental stressor.” The innovation of this study also resides in constructing a multi-dimensional integrated MPA research framework, integrating multiple factors including individual psychological traits, growth experiences, educational environment, and cultural background, which broadens the exploration dimensions of existing research.

Based on the research findings, this study offers clear practical implications for the identification, prevention, and intervention of MPA in higher music education. At the individual level, educators should guide students to foster appropriate self-efficacy and outcome expectations, help them overcome cognitive biases associated with being constrained by perfectionism and others' evaluations, and strengthen their self-regulatory capacity. At the behavioral level, the practice instruction model should be optimized, prioritizing effective practice over prolonged practice. Educators should guide students to formulate personalized practice plans, encourage them to transform anxiety into performance motivation, and develop positive behavioral coping strategies.

To promote the modernization of piano pedagogy in Chinese higher music education, targeted curriculum reforms can be implemented. Traditional emphasis on technical skill training may be combined with mental and physical regulation training. For example, Tai Chi can be included as a supplementary practice to help students improve breathing control, physical relaxation, and emotional stability. Arousal reappraisal can also be introduced into regular teaching, guiding students to view anxiety as a form of performance-enhancing arousal rather than a destructive symptom. These culturally adaptive and psychologically informed practices can help students establish long-term, effective strategies for managing MPA.

At the environmental level, a supportive educational climate should be established. Teachers need to adjust their high-pressure teaching approaches and offer more emotional support and experience sharing. Universities can set up psychological intervention mechanisms to deliver professional psychological support for tertiary student pianists. Meanwhile, educators should advise families to adopt reasonable music education concepts, reduce utilitarian expectations, and mitigate the environmental pressure faced by tertiary student pianists.

## 6 Research limitations and future prospects

This study has certain limitations. Firstly, although the sample size has reached data saturation, it is only sourced from 10 Chinese higher education institutions, and the geographical representativeness of the sample is limited. However, there are relatively few studies related to music performance anxiety (MPA) in China, and qualitative studies as well as studies related to student musicians are even more lacking. This has led to the long-term neglect of the mental health problems of student musicians, including their MPA-related experiences. This study can serve as a preliminary research to encourage more people to pay attention to the physical and mental health issues of student musicians.

Future research can expand the sample size to cover different regions and universities of different levels, and further verify the universality of the research results. In addition, although the study has focused on the influence of cultural background, it has not directly compared the MPA-related experiences among Chinese and tertiary student pianists from other countries. Future cross-cultural comparative studies can be conducted to systematically and comprehensively investigate the phenomena related to MPA among student pianists, as well as the risk factors and coping strategies of MPA. Moreover, future research can combine quantitative research methods to quantitatively verify the risk factors and coping strategies revealed by this study, achieving the complementarity of qualitative and quantitative research and enhancing the persuasiveness of the research conclusions.

## 7 Conclusion

In conclusion, based on the Social Cognitive Theory (SCT) theoretical framework and combined with the descriptive phenomenological method, this study conducted an in-depth exploration of the MPA-related experiences of student pianists in Chinese higher education institutions. It not only revealed in detail the personal, behavioral and environmental risk factors and coping strategies for MPA, but also identified several new findings and innovative insights with local characteristics. The research results have not only enriched the theoretical system and cross-cultural perspective of MPA research, but also provided practical and feasible paths for the identification and intervention of MPA in China's local music education. This study has expanded the application of SCT in the field of music education psychology, providing a new analytical framework and empirical reference for subsequent relevant research. At the same time, the risk factors and coping strategies identified in this study also offer scientific guidance for higher music educators, families and related practitioners, and hold reference value for advancing the mental health education and professional development of tertiary student pianists.

## Data Availability

The raw data supporting the conclusions of this article will be made available by the authors, without undue reservation.
