# Supplementary material for: Risk factors and coping strategies of music performance anxiety among student pianists in higher education: a phenomenological research perspective
Source: Front Psychol. 2026 Jun 25;17:1804936. doi: 10.3389/fpsyg.2026.1804936 (PMC13386414; doi:10.3389/fpsyg.2026.1804936)
Supplement: Supplementary file 1 [file Data_Sheet_1.PDF]

Supplementary Material 1 - Interview Guide

Part 1. Personal Factor

1. When and under what circumstances did you discover that you had MPA?

2. How did you feel physically and mentally when experiencing MPA? Were there any related changes?

3. What do you think are the personal factors that led to your suffering from MPA?

4. Do you have any strategies or methods for dealing with MPA on your own?

Part 2. Behaviour Factor

1. Which behaviors do you think have directly or indirectly contributed to your experience of MPA?

2. Which behaviors do you think prove helpful for alleviating your experience of MPA?

3. Do you have any expectations for improving your piano performing ability or alleviating your MPA? Will these expectations exert any influence on your subsequent behaviors?

4. Can you share your experiences of managing and coping with MPA?

Part 3. Environment Factor

1. What objective environmental factors do you think led to your experience of MPA?

2. Do you think the social environment can cause your experience of MPA (such as social relationships and social evaluations)?

3. After suffering from MPA, have you been able to receive help from the social environment or other individuals?
