# Supplementary material for: Risk factors and coping strategies of music performance anxiety among student pianists in higher education: a phenomenological research perspective
Source: Front Psychol. 2026 Jun 25;17:1804936. doi: 10.3389/fpsyg.2026.1804936 (PMC13386414; doi:10.3389/fpsyg.2026.1804936)
Supplement: Supplementary file 2 [file Data_Sheet_2.PDF]

Supplementary Material 2 - Consolidated Criteria for Reporting Qualitative research (COREQ) Checklist

A checklist of items that should be included in reports of qualitative research. You must report the page number in your manuscript where you consider each of the items listed in this checklist. If you have not included this information, either revise your manuscript accordingly before submitting or note N/A.

Topic	Item No.	Guide Questions/Description	Reported on Page No.
**Domain 1: Research team and reflexivity**			
Personal characteristics			
Interviewer/facilitator	1	Which author/s conducted the interview or focus group?	First author
Credentials	2	What were the researcher's credentials? E.g. PhD, MD	PhD
Occupation	3	What was their occupation at the time of the study?	University lecturer
Gender	4	Was the researcher male or female?	Two Female and one male
Experience and training	5	What experience or training did the researcher have?	The researchers have systematically studied qualitative research methods and music Psychology. They are also pianists and piano teachers.
Relationship with participants			
Relationship established	6	Was a relationship established prior to study commencement?	In order to avoid subjectivity affecting the results of the study, researchers and

			participants did not establish any close relationship.
Participant knowledge of the interviewer	7	What did the participants know about the researcher?e.g. personal goals, reasons for doing the research	Participants were informed of the plan, goals, purpose, and data collection methods of the study. Participants also learned basic demographic information about the researchers.
Interviewer characteristics	8	What characteristics were reported about the interviewer/facilitator? e.g. Bias, assumptions, reasons and interests in the research topic	The interviewer is a pianist and tertiary piano teacher, as well as a MPA topic researcher. She has a strong research interest and desire in the psychological problems and health issues of student pianists.
**Domain 2: Study design**			
Theoretical framework			
Methodological orientation and Theory	9	What methodological orientation was stated to underpin the study? e.g. grounded theory, discourse analysis, ethnography, phenomenology,<br><br>content analysis	Descriptive Phenomenology
Participant selection			
Sampling	10	How were participants selected?e.g. purposive, convenience,<br>consecutive, snowball	Purposive
Method of approach	11	How were participants approached?e.g. face-to-face, telephone, mail, email	Face-to-face
Sample size	12	How many participants were in the study?	29 tertiary student pianists

Non-participation	13	How many people refused to participate or dropped out? Reasons?	No one
Setting			
Setting of data collection	14	Where was the data collected?e.g. home, clinic, workplace	Digital piano classroom, music room, school meeting room and coffee shop
Presence of non-participants	15	Was anyone else present besides the participants and researchers?	Nobody
Description of sample	16	What are the important characteristics of the sample? e.g. demographic data, date	Lived experiences with MPA
Data collection			
Interview guide	17	Were questions, prompts, guides provided by the authors? Was it pilot tested?	The researchers provided interview guides and conducted a pilot study with for pianists in addition to the participants.
Repeat interviews	18	Were repeat interviews carried out? If yes, how many?	No
Audio/visual recording	19	Did the research use audio or visual recording to collect the data?	Used audio
Field notes	20	Were field notes made during and/or after the interview or focus group?	After
Duration	21	What was the duration of the interviews or focus group?	Interviews: 36-79 minutes<br>Focus group discussion: 97 minutes and 83 minutes
Data saturation	22	Was data saturation discussed?	Yes
Transcripts returned	23	Were transcripts returned to participants for comment and/or	Yes
